# Prevalence, Predictors, and Treatment of Impostor Syndrome: a Systematic Review

**DOI:** 10.1007/s11606-019-05364-1

**Published:** 2019-12-17

**Authors:** Dena M. Bravata, Sharon A. Watts, Autumn L. Keefer, Divya K. Madhusudhan, Katie T. Taylor, Dani M. Clark, Ross S. Nelson, Kevin O. Cokley, Heather K. Hagg

**Affiliations:** 1 Center for Primary Care and Outcomes Research, Stanford University School of Medicine, Stanford, CA USA; 2 Crossover Health, San Clemente, CA USA; 3 Untold Content, Cincinnati, OH USA; 4 Welleo Health, San Francisco, CA USA; 5 University of Texas at Austin, Austin, TX USA

**Keywords:** impostor syndrome, behavioral health, occupational health

## Abstract

**Background:**

Impostor syndrome is increasingly presented in the media and lay literature as a key behavioral health condition impairing professional performance and contributing to burnout. However, there is no published review of the evidence to guide the diagnosis or treatment of patients presenting with impostor syndrome.

**Purpose:**

To evaluate the evidence on the prevalence, predictors, comorbidities, and treatment of impostor syndrome.

**Data Sources:**

Medline, Embase, and PsycINFO (January 1966 to May 2018) and bibliographies of retrieved articles.

**Study Selection:**

English-language reports of evaluations of the prevalence, predictors, comorbidities, or treatment of impostor syndrome.

**Data Extraction:**

Two independent investigators extracted data on study variables (e.g., study methodology, treatments provided); participant variables (e.g., demographics, professional setting); diagnostic tools used, outcome variables (e.g., workplace performance, reductions in comorbid conditions); and pre-defined quality variables (e.g., human subjects approval, response rates reported).

**Data Synthesis:**

In total, 62 studies of 14,161 participants met the inclusion criteria (half were published in the past 6 years). Prevalence rates of impostor syndrome varied widely from 9 to 82% largely depending on the screening tool and cutoff used to assess symptoms and were particularly high among ethnic minority groups. Impostor syndrome was common among both men and women and across a range of age groups (adolescents to late-stage professionals). Impostor syndrome is often comorbid with depression and anxiety and is associated with impaired job performance, job satisfaction, and burnout among various employee populations including clinicians. No published studies evaluated treatments for this condition.

**Limitations:**

Studies were heterogeneous; publication bias may be present.

**Conclusions:**

Clinicians and employers should be mindful of the prevalence of impostor syndrome among professional populations and take steps to assess for impostor feelings and common comorbidities. Future research should include evaluations of treatments to mitigate impostor symptoms and its common comorbidities.

## INTRODUCTION

Impostor syndrome (also known as impostor phenomenon, fraud syndrome, perceived fraudulence, or impostor experience) describes high-achieving individuals who, despite their objective successes, fail to internalize their accomplishments and have persistent self-doubt and fear of being exposed as a fraud or impostor.^1^ People with impostor syndrome struggle with accurately attributing their performance to their actual competence (i.e., they attribute successes to external factors such as luck or receiving help from others and attribute setbacks as evidence of their professional inadequacy).^2^ Psychologists Clance and Imes first described impostor phenomenon in 1978,^2^ and it came to widespread public attention after Clance’s 1985 book.^3^ Clance originally identified the syndrome among high-achieving professional women, but more recent research has documented these feelings of inadequacy among men and women, in many professional settings, and among multiple ethnic and racial groups.^4, 5^

Impostor syndrome is not a recognized psychiatric disorder: It is not featured in the American Psychiatric Association’s Diagnostic and Statistical Manual^6^ nor is it listed as a diagnosis in the International Classification of Diseases, Tenth Revision (ICD-10).^7^ Outside the academic literature, impostor syndrome has become widely discussed, especially in the context of achievement in the workplace. Perhaps because it is not an officially recognized clinical diagnosis, despite the large peer review and lay literature, although there has been a qualitative review,^8^ there has never been a published systematic review of the literature on impostor syndrome. Thus, clinicians lack evidence on the prevalence, comorbidities, and best practices for diagnosing and treating impostor syndrome. Moreover, its actual effects on professional performance and burnout both among healthcare professionals and others are not known.

The purpose of this study was to critically evaluate the published literature on impostor syndrome—specifically to evaluate the prevalence of impostor syndrome in employed populations and characterize its relationship to workplace performance and burnout, characterize common comorbidities, and determine the most effective treatments for populations suffering from impostor symptoms.

## METHODS

### Data Sources and Searches

We developed search strategies for three databases: Medline, Embase, and PsycINFO for citations dated January 1966 to May 2018. We used search terms such as *Imposter Syndrome* and *Impostor Phenomenon* (Appendix). We also reviewed bibliographies of retrieved articles to obtain additional citations.

### Study Selection

We considered peer-reviewed studies eligible for inclusion if they reported data on the prevalence, comorbidities, or treatment of impostor syndrome. We excluded studies that were only reported as dissertations, validations of scales to identify impostor syndrome, described dementia- or delirium-based syndromes, or reported cases of legal fraud, impostor drugs, Munchausen’s, or Munchausen’s by proxy.

### Data Extraction and Quality Assessment

Two authors independently abstracted five types of data from each of the included studies onto pre-tested data collection forms: study variables (e.g., purpose of the study, study methodology, treatments provided); participant variables (e.g., demographics, professional setting); diagnostic tools used to assess for impostor symptoms, outcome variables (e.g., workplace performance, reductions in comorbid conditions); and pre-defined quality variables (e.g., human subjects approval, response rates reported). We resolved abstraction discrepancies by repeated review and discussion. If two or more studies presented the same data from a single patient population, we included these data only once in our analyses.

### Lay Literature Search

While performing background literature searches using general Internet search engines, we noted an abundance of non-peer-reviewed literature on impostor syndrome. Thus, we undertook a literature search to assess lay interest in the topic of impostor syndrome. We first used the search engine optimization tool Moz to evaluate the key terms used by authors of Internet articles on impostor syndrome. The term *Imposter Syndrome* was almost exclusively used (“Imposter Syndrome Test” was also used), whereas *Impostor Phenomenon* was never used. We then used the content curation and analysis tool BuzzSumo to evaluate the number of Internet articles indexed with the term “Imposter Syndrome” published between March 28, 2018, to March 28, 2019, and to measure the number of times these articles were shared, liked, or commented upon on social media platforms Facebook, Pinterest, Reddit, and Twitter.

### Data Synthesis and Analysis

Given the heterogeneity of the included studies, we summarized the populations assessed, diagnostic tools used, and reported prevalence, comorbidities, and any treatments provided qualitatively. The datasets generated during data collection and analysis are available from the corresponding author on reasonable request.

### Role of Funding Source

This work was funded by Crossover Health, a provider of employer-sponsored health clinics. The funder had no role in this study’s design, conduct, or reporting.

## RESULTS

**Figure 1:**
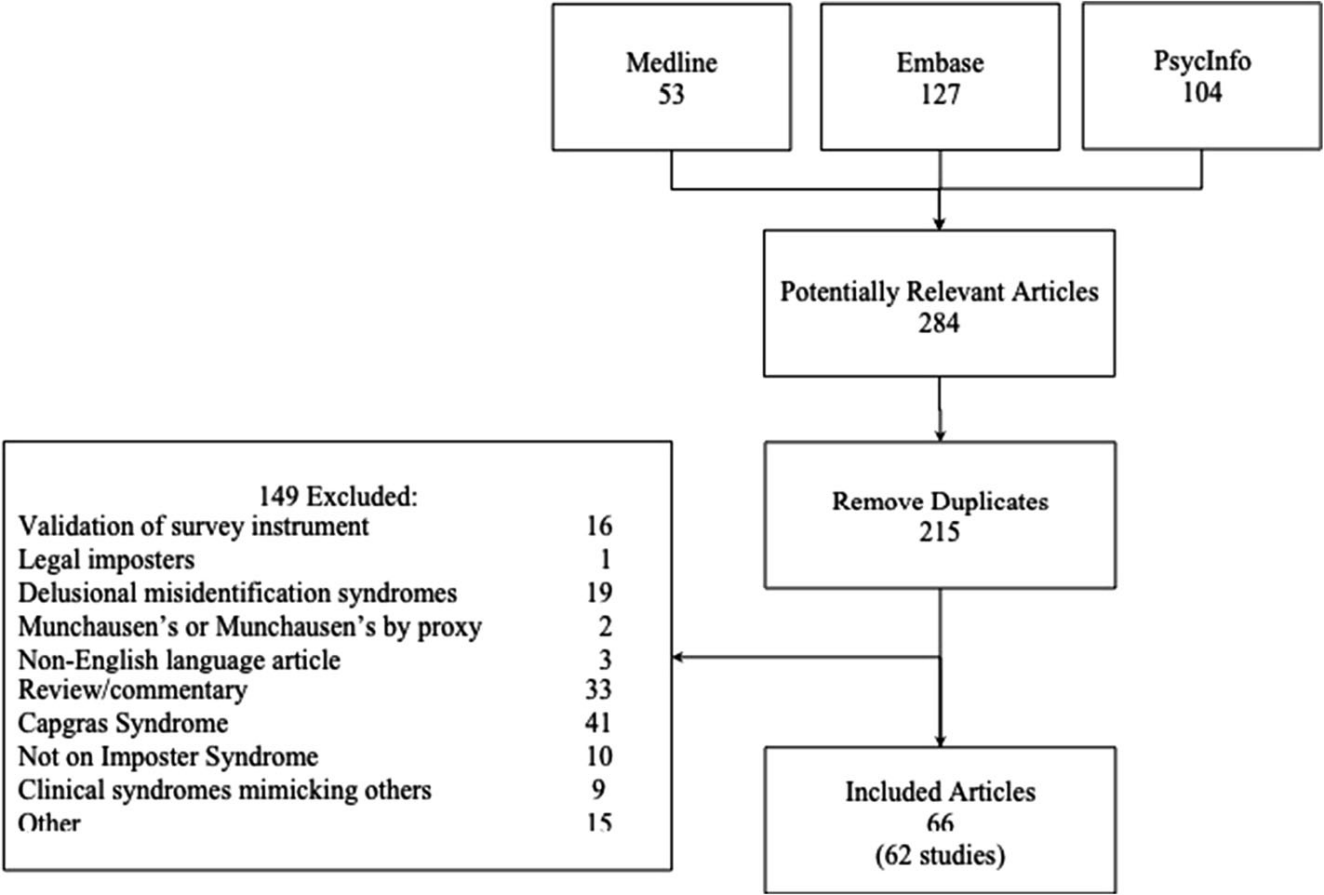
Search results. Presents our search strategies and results.

Our searches identified 284 titles of potentially relevant peer-reviewed articles (Fig. 1). After synthesizing the data from multiple reports on the same set of participants, 66 articles describing 62 studies with 14,161 participants met our inclusion criteria (Table 1).

**Table 1.** Study Characteristics

**Reference (Year of publication)**	**Country**	**Study Purpose**	**Population**	**Sample Size/Response Rate (RR)**	**Gender (% women)**	**Age (mean)**	**Study Design**	**Main Findings**
Ares, T.L. (2018)(9)	USA	Evaluate the prevalence of IS in early career clinical nurse specialists	Clinical nurse specialists (CNS)	68 RR- 61.9%	94%	43% was 30-39 yrs	Cross sectional survey	Prevalence: 74.6%. Prevalence of IP was not different in CNSs who were employed vs those who were unemployed. IP was not predicted on the basis of perceived preparedness for CNS practice, experiential preparation for practice, or years of leadership experience.
Austin et al. (2009) (10)	USA	Explore the mediating effects of impostorism on the association between survivor guilt and depression	Black/African-American college undergrads	97 RR -19.58%	72%	24yrs	Cross sectional survey	Prevalence: Not Stated. Survivor guilt is a significant positive predictor of depression. Participants who reported greater levels of impostor suffering also reported greater degrees of survivor guilt feelings. Impostor feelings significantly predicted depression. There were no gender differences in IP.
Bechtoldt (2015)(11)	Germany	Analyzes how impostorism affects a specific component of leadership behavior, which is task delegation	Managers of various industries	190 RR – 41.5%	35%	45yrs	Cross sectional survey	Prevalence: Not Stated. The more strongly managers lacked confidence in their own abilities and perceived themselves as impostors, the more inclined they were to also assign challenging tasks to the insecure male and female employees. There was no significant association between managers' impostorism and their decisions on delegating these tasks to the self-confident employees.
Bernard et al. (2017)(12) (2018)(13)	USA	Examine the association between racial discrimination and the impostor phenomenon	African American college students	157 RR – not stated	68%	Cohort 1-19yrs Cohort 2-18yrs	Cross sectional survey	Prevalence: Not Stated. Women reporting high frequency of racial discrimination but lower levels of distress resulting from racial discrimination had higher levels of IP.
Brauer & Proyer (2017)(14)	Germany	Evaluate the association of impostor phenomenon and positive coping skills such as playfulness	Sample 1 – Psychology students Sample 2 – Working professionals	Sample 1-244 RR – not stated. Sample 2-222 RR– not stated	Sample 1-63% Sample 2-63%	Sample 1-23yrs Sample 2 – 37yrs	Cross sectional survey	Prevalence: Not Stated. Among both students and working professionals, playfulness (an important characteristic of resilience) was negatively correlated with IP. The authors report mixed correlations between different types of playfulness among students and working professionals with and without IP. Age was significantly negatively correlated with IP among professionals (p<0.001) but not for students.
Byrnes & Lester (1995)(15)	USA	Assess locus of control in people with imposter syndrome	Accountants and teachers	60 RR – not stated	63%	38yrs	Cross sectional survey	Prevalence: Not Stated. Individuals believing more strongly in an external locus of control were more likely to feel like an imposter.
Caselman et al. (2005)(16)	USA	Evaluate gender, global self-worth, social support, and self-concept as predictors of IP among adolescents	High school juniors and seniors	136 RR – not stated	52%	Not Stated	Cross sectional survey	Prevalence: Not Stated. Symptoms of IP are found among adolescents. Global self worth, parent support, classmate support, teacher support, friend support, sociability, competence and dependability were all significantly inversely correlated with IP (p<0.01). There was no gender difference in mean IP scores; however, the specific types of support that were most predictive of IP for boys and girls varied.
Castro, Jones & Mirsalimi, (2004)(17)	USA	Evaluate the prevalence of imposter syndrome among individuals who were parentified as children	Psychology graduate students	213 RR – 27.5%	85%	31yrs	Cross sectional survey	Prevalence: 80% reported at least moderate levels of impostor feelings and 30% endorsed significant impostor feelings; Mean IP score (Clance 20 item scale) was 55.19. Further,Caucasians endorsed more IP symptoms than African Americans. Parentification in childhood was highly correlated with impostor phenomenon.
Chae et al. (1995)(18)	Korea	Assess the prevalence of IP in Korea	Catholics	654 RR – not stated	49%	34yrs	Cross-sectional cohort	Prevalence: 39% using a cutoff of 58 on CIPS and 24% using a cutoff of 62 on CIPS; Mean IP score (Clance 20 item scale) was 56.2 with Standard Deviation of 9.7. IP high scorers are anxious, depressed, emotionally unstable, prone to psychological distress, and negative affect. They tend to be less competent and to procrastinate, are easily discouraged, and tend to avoid hard work. Interpersonal style is glum, detached, uncommunicative, aloof, and skeptical. They are introverted and suspicious of the motives of others. No association with gender or education. However, younger women had higher IP scores.)
Chayer & Bouffard (2010)(19)	Canada	Evaluate the association of the use of social comparisons with IP	5th and 6th grade students	740 RR – not stated	50%	10-12 yrs	Cross sectional survey	Prevalence: Not Stated; Mean IP score (Likert-type scale, rated on 4-point scale) was 1.6. There was no difference by grade or gender in imposter feelings. 20% of the students did not feel like impostors at all; 80% felt like impostors to some degree, and 3% said they felt "quite like" or "just like" impostors. Boys reported a higher tendency to use social comparison than girls and a greater use of downward contrast than girls.
Christensen et al. (2016)(20)	Australia, New Zealand, UK	Examine the prevalence of IS in final year nursing students	Nursing Students	223 RR - Aus -23.9% NZ – 78.4% UK -86.6%	Not Stated	Not Stated	Cross sectional survey	Prevalence: 45.1 % had moderate imposter phenomenon, 33.4% were classified as frequently having imposter feelings, and 8.3% described as often experiencing intense IS experiences. A positive weak correlation between IP and preparedness for practice was found.
Cokley et al. (2013)(21)	USA	Examine differences related to minority stress status, imposter feelings, and mental health in ethnic minority students	College students	240 RR -not stated	62%	21yrs	Cross sectional survey	Prevalence: Not Stated; Mean IP scores (Clance 20 item scale, rated on 5-point scale) were Asian Americans (AMAs) 3.09 vs Latino/a Americans (LAs) 2.80 vs African Americans (AAs) was 2.56. AAs reported significantly more group stress, race-related stress, and environmental stress than LAs and ASAs. There were no differences by gender on IP. ASAs reported significantly higher imposter feelings than AAs or LAs. There were no differences found in IP by AA and LA. Imposter feelings were significantly positively correlated with minority status stress and negatively correlated with psychological wellbeing. IP was a stronger predictor of mental health than minority status stress.
Cokley et al. (2017)(22)	USA	Evaluate the association between imposter syndrome, perceived discrimination, and mental health among minority students.	College students	322 RR – not stated	70%	21yrs	Cross sectional survey	Prevalence: Not Stated; Mean IP scores (Clance 20 item scale, rated on 5-point scale) were Asian Americans (ASAs) 3.19, SD .64 vs Latino/a Americans (LAs) 3.00, SD .76 vs African Americans (AAs) 2.99, SD .77. AAs reported higher perceived discrimination than ASAs and LAs. There were no differences in imposter feelings by racial/ethnic group. Among AAs and LAs imposter feelings were not predictive of depression but were for anxiety. Among ASAs, imposter feelings predicted both anxiety and depression.
Cokley et al. (2015)(23)	USA	Examine the relationship of gender stigma consciousness, impostor phenomenon and academic self-concept	African American College students	491 RR – 97.2%	70%	21yrs	Cross sectional survey	Prevalence: Not Stated; Mean IP scores (Clance 20 item scale, rated on 5-point scale) were male was 2.94, SD .69 vs female 3.95, SD .74. IP was correlated with gender stigma consciousness for both women and men. IP and minority stress were significantly negatively correlated with academic self-concept. There was no association between IP and grade point average.
Cowman & Ferrari (2002)(24)	USA	Evaluate the association of self-reported imposter tendencies and self-handicapping tendencies and shame and guilt affect	College students	436 RR – not stated	68%	Not Stated	Cross sectional survey	Prevalence: Not Stated; No gender differences in mean IP scores (Clance 20 item scale, rated on 5-point scale). Mean CIP for group: 59.25 (SD 13.86). Imposter tendencies were significantly predicted by behavioral self-handicapping, shame and guilt affect (p<0.05).
Cozzarelli & Major (1990)(25)	USA	Compare the psychological responses of impostors and non-impostors to a midterm exam	College students	137 RR – not stated	62%	Not Stated	Prospective cohort study	Prevalence: Not Stated; Mean IP score (Clance 20 item scale) was 65.71. Students were given questionnaires at 3 times to assess pre- vs post- mid-term status. Authors used the median of the CIP scores to distinguish imposters from non-imposters. Impostor feelings were significantly related to pessimism and self-esteem. Imposters expected to perform worse and were more anxious about the exam than non-imposters, but actual grades did not significantly differ. Impostors were significantly more likely to make attributions to bad luck and low ability regardless of outcome than were non-impostors. Impostors were significantly less satisfied with their grades after failure than were non-impostors, but there were no differences between groups in satisfaction after success. Imposters had lower post exam self-esteem than non-imposters.
Crawford et al. (2016)(26)	USA	Examine association between the IP and work-to-family conflict	Employees of community college	92 RR – 25.27%	64%	47yrs	Cohort analysis with 3 data collection intervals	Prevalence: Not Stated; Mean IP score (Clance 20 item scale) was 2.57, SD .62. Emotional exhaustion is a mediating mechanism in the relationship between the IP and Work Family Conflict (WFC). Perceived Organizational Support (POS) is a moderator of this relationship. When employees perceive high levels of POS, the relationship between IP and WFC is minimized.
Cromwell et al. (1990) (27)	USA	To evaluate the effects of gender, grade, level, GPA, and personality characteristics on the prevalence of IP among high school students	English honors students in grades 9-12	104 RR – 18.4%	64.40%	Range: 14-18yrs	Cross sectional survey	Prevalence: 20.9% . Students were divided into two groups based on IP score (above 39 = imposters; under 39 = non-imposters). Non-imposters had a mean IP score of 28.73, SD .6.7 and imposters had a mean IP score of 45.7, SD 5.1. No students scored above the clinical cutoff of 62. There were no differences in impostorism by gender. Imposter feelings were associated with perfectionism and irrational beliefs.
Cusack et al. (2013)(28)	USA	Evaluate the effects of gender and other variables on IP	College students	506 RR – not stated	79%	21yrs	Cross sectional survey	Prevalence: Not Stated; Mean IP Score (Clance 20 item scale) was 58.68, SD 13.87. Women were significantly more likely to report impostor beliefs than men. Mental health, perfectionism, and test anxiety were significantly related to impostor beliefs, whereas low self-esteem was not related to the IP.
Ewing et al. (1996) (29)	USA	Explore the relationship between racial identity, world view, graduate school environment and IP	African American Graduate Students	103 RR – 26%	70%	31yr	Cross sectional survey	Prevalence: Not stated. Standard multiple regression analysis indicated that a model that included Belief Systems Analysis Scale and Racial Identity Attitudes Scale scores contributed to significant proportion of variance in IP scores F (5,94) = 3.48. R^2^ = .16, p ≤ .01. Worldview was a better predictor of susceptibility to the imposter phenomenon than racial identity attitudes. When combined with academic self-concept, racial identity attitudes significantly contributed to predicting imposter feelings. Authors point out that imposter syndrome scales have not been normed on Non-White populations.
Ferrari, J.R. (2005)(30)	USA	Explore association of academic dishonesty with IP	College students	124 RR – not stated	74%	21yrs	Cross sectional survey	Prevalence: 25.8%; Mean IP score (Clance 20 item scale) was 54.24, SD 13.27. Students with imposter feelings were significantly less likely to cheat on examinations or to plagiarize in written assignments than students without imposter feelings.
Ferrari & Thompson (2006)(31)	USA Australia	Examined impostor fears, self-handicapping and self-presentational concerns	College students Study 1 –in USA Study 2 - in Australia	Study 1- 165 RR -not stated Study 2 – 72 RR – not stated	Study 1 - 68.4% Study 2 - 100%	Study 1 - 21yrs Study 2 -21yrs	Study 1 - Cross sectional Study 2 - Experimental	Prevalence: Not stated; Mean IP score (Clance 20 item scale) was 55.75, SD 13.97 and 62.68, SD 12.54 in Study 1 and 2 respectively. In Study 1, imposter fears were significantly related to social desirability, perfectionistic cognitions, and non-display of imperfection to others. In Study 2, women were exposed either to face-saving failure (failure that did not indicate low ability, thereby assuaging self-presentational concerns), humiliating failure (where no mitigating excuse for poor performance was available), or success. Following humiliating failure, participants high in impostor fears claimed more handicaps than those low in imposter fears. However, when provided with a face-saving excuse, these participant groups did not differ in their propensity to claim handicaps.
Fried - Buschalter (1997)(32)	USA	Investigate gender differences in fear of success, fear of failure, and the IP	Employees - Marketing managers	104 RR – 92.9%	49%	35yrs	Cross sectional survey	Prevalence: Not Stated; Mean IP score (14 item Harvey Scale) was 43.16, SD 12.53. Women were significantly (p < .05) higher than the men on fear of success, but there were no significant gender differences on fear of failure or the IP. Among both female and male managers, significant positive correlations were observed between fear of failure and the IP.
Ghorbanshirodi (2012)(33)	Iran	Evaluate the relationship between self-esteem, emotional intelligence, and imposter feelings	Medical students	200 RR – not stated	Not stated	Not stated	Cross sectional survey	Prevalence: Not Stated. Self-esteem was negatively correlated with imposter syndrome (p=0.0001). One aspect of emotional intelligence (utilization of emotion) was significantly associated with imposter syndrome. There was a significant gender difference in the association of emotional intelligence and imposter symptoms.
Gibson-Beverly & Schwartz (2008)(34)	USA	Evaluate whether prior parental attachment and perceptions of entitlement are predictors of IP	Female graduate students	170 RR – not stated	100%	34yrs	Cross sectional survey	Prevalence: Not Stated; Mean IP score (20 item Clance Scale) was 54.37, SD 13.22. Anxious attachment and entitlement were positively correlated with the Clance IP Scale (p < .01 for both).
Hayes & Davis (1993)(35)	USA	To investigate the relationships between interpersonal flexibility, Type A behavior, and impostor characteristics	Undergraduate students	83 RR – not stated	71%	Men – 22yrs Women - 21yrs	Cross sectional survey	Prevalence: Not Stated. Interpersonal flexibility was negatively related to impostor characteristics for both men and women. Type A and impostor characteristics were negatively related for men, they were positively related for women. The results of these analyses indicated that the men and women did not differ with respect to interpersonal flexibility (P > .08) and Type A characteristics (P =.53). However, women had significantly higher scores on the impostor test (P < .02).
Henning et al. (1998)(36)	USA	Examine the prevalence and severity of psychological distress and the IP in health profession students	Graduate students (in health professions)	477 RR – 48%	53%	26yrs	Cross sectional survey	Prevalence: 30.2%; Mean IP scores (Clance 20 item scale) were 57.83, SD 14.89 for women vs 52. 08, SD 13.03 for men. 27% of medical, dental, nursing and pharmacy students reported current symptoms of psychological distress. 30% of the students had IP using a cut off of 62 on the CIPS. Significantly more women than men met criteria for IP (37.8% vs. 22%, P < 0.001). Psychological distress was significantly correlated with feelings of IP.
Hutchins & Rainbolt (2016) (37) Hutchins et al. (2018)(38)	USA	Evaluate emotional exhaustion and job satisfaction among faculty with IP	Academic Faculty	Study 1 – 16 Study 2 -310 RR – 17%	63% 59%	Not Stated	Cross-sectional survey and semi-structured interview	Prevalence: Not Stated; Mean IP score (Clance 20 item Scale) was 2.81 and 2.55 in sample 1 and sample 2 respectively. IP was positively related to emotional exhaustion (p < .01) and negatively related to job satisfaction (p < .01). Men and women differ in their coping strategies to manage IP.
Jöstl et al. (2012)(39)	Austria	Evaluate gender differences in both IP and research self-efficacy of doctoral students	Graduate students	631 RR – not stated	62%	32yrs	Cross sectional survey	Prevalence: 82% (at least low levels of IP) with 33% of the sample reporting symptoms of IP Women reported higher scores on the IP Scale and lower levels of research self-efficacy than men. Women had greater fear of success and fear of failure, and lower self-esteem than men. Faculty members reported higher levels of both the IP and research self-efficacy than non-faculty members.
Kamarzarrin et al. (2013)(40)	Iran	Evaluate the association between self esteem and imposterism	Physicians	65 RR – not stated	46%	Not Stated	Cross sectional survey	Prevalence: Not Stated. Self esteem is negatively correlated with imposterism (p=0.01). There was no difference in the association of self esteem and imposterism between men and women.
Kananifar et al. (2015)(41)	Iran	Evaluate the relationships between IP and mental health	Esfahan university Students	400 RR – not stated	Not Stated	Not Stated	Cross sectional survey	Prevalence: Not Stated. They found significant positive correlations between IP and somatic symptoms, anxiety and insomnia, social dysfunctions, and depression.
King & Cooley (1995)(42)	USA	Evaluation the association between IP and family achievement orientation and achievement-related behaviors	College students	127 RR – not stated	59%	19yrs	Cross sectional survey	Prevalence: Not Stated; Mean IP scores (Clance 20 item scale) were 59.47, SD 14.59 women vs 53.40, SD 11.15 men. No gender differences in family achievement orientation, but significantly higher CIPS scores among females; A positive correlation was found between IP and family achievement orientation. No sig differences in grade point average by gender. Higher levels of IP were found among females with IP but not for males. No gender differences in number of hours worked per week. Greater number of hours worked outside the classroom on academics was significantly correlated with IP for females, but not for males.
Kumar & Jagacinski (2006)(43)	USA	Evaluate the relationship between imposter fears and achievement goals	Psychology undergraduate students	135 RR – not stated	31%	19yrs	Cross sectional survey	Prevalence: Not Stated. Women reported greater imposter fears and were higher in ability-avoid goals than men. Among women, imposter fears were significantly associated with endorsement of the entity viewpoint p < .001. For both men and women, imposter fears were positively related to test anxiety and negatively related to confidence in intelligence.
LaDonna et al. (2018)(44)	Canada	Identify strategies to support physicians who struggle with underperformance	Physicians	28 RR – 2.8%	36%	Not Stated	Qualitative Study (Semi -structured Interviews)	Prevalence: Not Stated. Not all participants identified as imposters; instead, the authors consider IS as only occurring at the extreme end of the spectrum of self-doubt. During residency, self-doubt is mostly around a pervasive concern that their medical skills and knowledge were not as good as they thought they were. For consultants, their insecurities suggested a fear that they were not as good as others thought they were.
Leary et al. (2000)(45)	USA	Study 1: Evaluate whether impostors believe others perceive them more positively than they perceive themselves. Study 2: Evaluate whether the characteristics attributed to impostors reflect interpersonal strategies Study 3: Explore whether 2 types of impostors exist—those who believe that they are not as good as other people think and those who falsely claim that others have overestimated them.	Undergraduate students	Study 1: 238 Study 2: 95 Study 3: 67 RR – not stated	Study 1: 50% Study 2: 49.47% Study 3: Not stated.	Study 1 range: 17-23yrs. Study 2 and 3: Not stated	Cross sectional survey	Prevalence: Not Stated. Study 1: Impostorism is more clearly a function of self-evaluations than of the discrepancy between self-evaluations and reflected appraisals. Study 2: No sig difference in impostorism by gender. High impostorism was negatively correlated with expected performance. When impostorism scores were split at the median, high impostors responded that they would score, on the average, in the 66^th^ percentile whereas low impostors indicated that they would score in the 75^th^ percentile. When participants thought their responses were public, high impostors expressed lower expectations regarding performance, reported that doing well on the test was less important, derogated the validity of the test, and expressed less satisfaction about the possibility of performing well. Study 3: Impostors indicated they would feel less good about performing well when their scores were public rather than private. Impostorism was negatively correlated with self-confidence and self esteem.
Legassie et al. (2008)(46)	Canada	Explore the prevalence and association between impostorism and burnout syndrome	Internal medicine residents	48 RR – 62.3%	52%	30yrs	Cross sectional survey	Prevalence: 43.8%; Mean IP score (Clance 20 item scale) was 61.2, 14.2 SD Impostorism and burnout syndrome were identified in 43.8% and 12.5% of residents, respectively. The mean raw score for CIPS responses was 61.2. Females (p= .03) and foreign medical graduates (p= .03) reported significantly higher CIS scores. A significant negative correlation was detected between raw scores on the personal accomplishment subscale and the CIPS (p=.04)
Leonhardt et al. (2017)(47) and Rohrmann et al (2016)(48)	Germany	Examine whether the IP is a homogeneous construct or whether different types of persons with IP can be distinguished on the basis of related characteristics	Professionals in leadership positions	242 RR – 55.14%	37%	44yrs	Cross sectional survey	Prevalence: Not Stated. Persons scoring high on the CIPS were more frequently employed in civil service than in the private sector. Those scoring high on IP could be clustered into two subgroups: "true imposters" (who displayed high stress and strain, negative self-evaluation, procrastination, and largely negative emotional experiences) and "strategic imposters" (with trait levels resembling those without imposter self-concept). This latter group are characterized by a form of deliberate self-presentation. CIP is highly positively correlated with work-related stress and strain, dysphoric mood, anxiety and is highly negatively correlated with indicators of positive self-evaluation. No association was found between imposter phenomenon and gender.
Lester & Moderski (1995)(49)	USA	Prevalence of IP in high school students	High school students	233 RR – not stated	41%	16yrs	Cross sectional survey	Prevalence: Not Stated. Correlations between impostorism were associated with a history of prior suicidal ideation and attempts (p<.001)—this correlation remained significant after being controlled for depression. Impostorism was also correlated significantly with psychoticism, neuroticism, irrational thinking, and manic and depressive tendencies (p<.05 for all). Age and gender were not associated with impostor scores.
Li et al. (2014)(50)	USA	Evaluate the association of caring and overprotective behaviors of parents to IP in their children	Undergraduate and graduate college students	506 RR – not stated	79%	21yrs	Cross sectional survey	Prevalence: Not Stated; Mean IP score (Clance 20 item scale) for men = 54.57, SD 9.58 vs women = 59.75, SD 14.61. Women reported significantly more impostor feelings than men (p < .001). For women, parental care was negatively related to impostor feelings (p < .001) and parental overprotection was positively related to impostor feelings (p < .001). For men, neither correlations were significant.
Lige et al (2017)(51) and Peteet et al. (2015)(52)	USA	Evaluate the relationships between racial identity, self-esteem, and IP	African American college students	112 RR – not stated	74%	Not Stated	Cross sectional survey	Prevalence: Not Stated; Mean IP score (Clance 20 item scale) was 54.48, SD 14.74 There was no significant correlation between CIPS and gender, family income, class standing, or racial identity. However, there was a significant association between IP and grade point average and low self-esteem Higher impostorism scores were found to predict higher psychological distress and lower self-esteem.
Matthews & Clance (1985)(53)	USA	Present the experience of patients cared for in their private practices	Professionals	41 RR – not stated	80%	Not Stated	Descriptive cohort analysis	Prevalence: Not Stated. The found no gender effect in the prevalence of imposter syndrome (p=0.05). The present a qualitative description of their experience in private practice caring for patients with imposter feelings and note the importance of validating patients’ doubts and fears, directly addressing fears of failure, and providing group therapy.
McClain et al. (2016)(54)	USA	Evaluate the association of minority stress status, IP, racial centrality, and ethnic identity on mental health among African American college students	College Undergraduates	218 RR – not stated	72%	21yrs	Cross sectional survey	Prevalence: Not Stated Minority stress and IP were significantly negatively related to mental health whereas ethnic identity and racial centrality were positively related to mental health. No significant gender differences were found
McElwee & Yurak (2007)(55)	USA	Evaluate the differences in affect and impression management styles between non-impostors, strategic impostors, and true impostors	College students (undergrad and graduate level)	Sample A- 124; Sample B- 125; RR – not stated	Sample A: 84%. Sample B: 64%	20yrs	Cross sectional survey	Prevalence: Not Stated. Impostorism is associated with greater levels of negative affect and lower levels of self-esteem and positive affect. Impostor scores were not predicted by low self-appraisals combined with high reflected appraisals, but instead were predicted only by low self-appraisals. Impostorism correlated positively with self-handicapping and negatively with the self-enhancing strategies. True impostors had lower self-esteem and more negative affect than did strategic impostors; however, they did not differ on positive affect.
McElwee et al. (2010)(56)	USA	Examine individuals' descriptions of IP episodes to identify their affective content and the situational and social antecedents	College undergrads	122 RR – not stated	74%	20yr	Cross sectional survey	Prevalence: Not Stated. Qualitative analyses yielded emergent themes in the narratives of students with impostorism including fear of excessive future expectations and positive affect as a result of the perceiver's view. Quantitative analyses, found that those scoring higher on IP scales reported more fear/distress and guilt/shame during IP episodes but did not differ on positive affect nor on hostility, compared to those scoring relatively low on the IP scales. IP scores were positively associated with the desire to correct the perceiver's impression, both at the time of the episode and at the time of survey completion.
McGregor et al. (2008)(57)	USA	Examine the relationship between the IP and depression	Liberal Arts college students	186 RR – not stated	62%	Not Stated	Cross sectional survey	Prevalence: Not Stated; Mean IP score (Clance 20 item scale) was 56.33, SD = 11.59. Women had higher IP scores than men (p=.003). However, there was no difference between men and women on the Beck depression Inventory. IP was highly correlated with depression scores.
Neureiter & Traut-Mattausch (2016)(58)	Study 1: Austria Study 2: Germany	Evaluate whether IP is a potential psychological barrier in the career development process.	Study 1: College undergrads Study 2: Airport employees	Study 1: 212 Study 2: 110 RR – not stated	Study 1: 70% Study 2: 50%	Study 1: 23yrs Study 2: 33yrs	Cross sectional survey	Prevalence: Not Stated. In both study 1 and 2: Impostor feelings were associated with fear of failure, fear of success, and low self-esteem. Participants who reported more impostor feelings reported less career planning, less career striving, and less motivation to lead
Neureiter & Traut-Mattausch (2016)(59)	Austria	Examine the IP’s impact on career optimism, career adaptability, knowledge of the job market, employee- and organizationally-relevant outcomes	Working professionals	238 RR – 66%	57%	Not Stated	Cross sectional survey	Prevalence: Not Stated. People with IP have less career planning, job satisfaction, and organizational citizenship behavior than those without IP. The authors conclude that to cover their perceived fraudulence, imposters are likely to work hard to fulfill the demands of in-role behavior according to their own high standards. As personal resources are restricted, this will result in less extra-role behaviors.
Neureiter & Traut-Mattausch (2017)(60)	Austria	Investigate how IP is related to career planning and occupational self-efficacy	University students	289 RR – not stated	75%	25yrs	Cross sectional survey	Prevalence: Not stated. IP is negatively correlated with planning, career exploration, and occupational self-efficacy (p < 0.001). Furthermore, the IP correlated highly significantly with three (concern, control, confidence) of the four dimensions of career adaptability resources (ps < 0.01).
Okoth et al. (1994)(61)	USA	Evaluate the association between general irrational thinking and imposter feelings in “disturbed” adolescents	Adolescents in a Federal Services day-care program	21 RR – not stated	62%	15yrs	Comparative evaluation	Prevalence: Not stated. “Disturbed” adolescents had higher impostor scores (p=0.01) but lower irrationality scores (p=0.04) than the normal high school students presented in Lester & Moderski.(49) Irrationality and imposter scores were correlated.
Oriel et al. (2004)(62)	USA	Evaluate the prevalence of impostor feelings in family medicine residents and whether impostor scores are associated with anxiety and depression.	Family medicine residents	185 RR – 73%	52%	33yrs	Cross-sectional survey	Prevalence: Men – 24% vs Women – 41%; Mean IP scores (Clance 20 item scale) were 54.3 men vs 58.5 women, (p 0.03). There were no gender differences in self-esteem, depression or state anxiety scores but women had higher trait anxiety (p 0.01). Impostor scores were correlated with depressive symptoms (p<0.0001), with trait and state anxiety (p<0.0001 for both) but not with years of training, age or marital status. Those with the highest impostor scores had the lowest self-esteem (p<0.0001). The impostors were more likely to worry that they will not be ready to practice after graduation. In multivariate analysis, when trait anxiety was controlled, gender no longer predicted impostor feelings but depression, trait anxiety and self-esteem remained statistically significant.
Patzak et al. (2017)(63)	Austria	Examine self-compassion as a potential resilience factor against the IP	College students	459 RR – 72%	69%	21yrs	Cross sectional survey	Prevalence: 9% Intense imposter feelings, 31% frequent imposter feelings, 46% moderate imposter feeling and 14% few imposter feelings. Fender and gender-role orientation was both statistically associated with the IP. Male students suffered less intensely from the IP than female students; masculine and androgynous students suffered less intensely from the IP than feminine students. Self-compassion was negatively correlated with IP; with increasing intensity of IP, self-compassion decreases. Self-compassion was found to mediate between gender-role orientation and the IP.
Peteet et al. (2015)(64)	USA	Examine the extent to which 1st-generation status, psychological well-being, and ethnic identity predict IP scores among high-achieving racial/minority undergraduates	African or Hispanic American undergraduate with a college GPA of > 3.0	161 RR – 14.6%	74%	Not Stated	Cross sectional survey	Prevalence: Not Stated; Mean IP score (Clance 20 item scale) was 50 SD ± 14.78. Results indicated overall moderate impostor feelings/experiences among the sample. Higher scores on the environmental mastery subscale of the well-being measure and the affirmation/belonging subscale of the ethnic identity measure were associated with decreased IP scores.
Robinson & Goodpaster (1991)(65)	USA	Examine the differences in IP among persons with alcoholic and non-alcoholic parents	College & Graduate students, members of Adult Children of Alcoholics	69 RR – not stated	Not Stated	Range – 19-51yrs	Cross sectional survey	Prevalence: Not Stated. IP was highest among the Adult Children of Alcoholics group and lowest among students with non-alcoholic parents.
Schubert & Bowker (2017) (66)	Canada	Examine the IP in relation to self-esteem	College undergrads	304 RR – not stated	75%	20yrs	Cross sectional survey	Prevalence: Not Stated; Mean IP score (Clance 20 item scale) was 62.45, SD = 13.10 Lower self-esteem was associated with higher impostor scores.
Selby & Mahoney (2002)(67)	USA	Examine the consequences of self-focused attention among self-described imposters and non-imposters who exhibited varying degrees of complexity in their self-systems	Graduate students	50	52%	28yrs	Experimental analysis (pre-post a specific mirror task)	Prevalence: 40%; Mean IP score (Clance 20 item scale) was 54.64, SD 12.20 Imposters’ galvanic skin responses (GSR) responses during the control condition (M=16.11, SD=9.17) were significantly lower during mirror 2 condition (19)=2.99,p=.008,but not different from their responses during mirror 1 condition, t(19)=.065,p=.52). Likewise, their GSR responses during mirror 1 condition were substantially lower than during the mirror 2 condition t(19)=5.23,p<.001. Imposters and Non imposters did not differ in average GSR responses during the Mirror 2 condition, t(48)= -.97,p=.397. However, imposters did report significantly lower self esteem scores after the experimental condition than non-imposters t(48) = -4.94,p<.001
September et al. (2001)(68)	Canada	Relate well-being to IP and gender role orientation	College students	379 RR – 23%	68%	22yrs	Cross Sectional survey	Prevalence: Not Stated. There were no differences in IP by gender or grade point average as measured by CIPS with a threshold of 62. High IPs scored lower in well-being and self- acceptance. Sig differences were found in IP by gender role orientation.
Sonnak & Towell (2001) (69)	UK	Investigate the role of perceived parental rearing style, parental background, self-esteem, mental health and demographic variables upon IP intensity	College students	107 RR – not stated	73%	26yrs	Cross sectional survey	Prevalence: 43%; Mean IP score (Clance 20 item scale) was 70.59, SD 6.16. Both greater degree of perceived parental control and lower levels of self-esteem were significant predictors of impostor fears. Parental care score, parental educational and occupational level and subject's mental health and demographic information did not show a significant relationship to impostor scores. A post-hoc regression analysis indicated, however, that in addition to parental protection, lower care and poorer mental health was significantly related to increasing levels of impostor scores and with subjects having attended private school reporting lower levels of impostor feelings. In addition, subjects classified as impostors were found to report significantly higher GHQ scores (poorer mental health) than non-impostors.
Thompson (1998)(70)	Australia	Clarify the affective and attributional behavior of impostors following success and failure feedback	College students (Psychology)	164 RR – 65%	77%	20yrs	Cross sectional survey	Prevalence: 48.8%; Mean IP score (Clance 20 item scale) was 73.05, SD 6.68. Using the 62-cutoff score, 48.8% of respondents were classified as impostors with 51.2% classified a non-impostor on CIPS. While a higher percentage of females (50.8%) relative to males (39%) were classified as impostors, this difference did not reach statistical significance. Age was negatively associated with impostor fears. Imposters felt greater humiliation and guilt after failure than success; there was no sig difference in these in either the success/failure condition for non-imposters. Imposters attributed poor performance to internal factors to a greater extent than non-imposters. Imposters reported lower academic and global self-esteem than non-imposters. There was no difference in GPA between imposters and non-imposters.
Thompson (2000)(71)	Australia	To assess the connection between impostor fears and perfectionistic concern over mistakes	College students (Psychology)	60 RR – not stated	82%	21yrs	Cross sectional survey	Prevalence: Not assessed in this study. Imposters reported less control (p <0.005), greater anxiety (p <0.002), more negative affect and greater concern over mistakes (p <0.01), than non-imposters when performing tasks.
Vergauwe (2015)(72)	Belgium	Examine the trait-relatedness of the IP and the potential impact of IP on relevant work attitudes and organizational citizenship behavior	White collar workers	201 RR – not stated	58%	36yrs	Cross-sectional survey	Prevalence: 20%; Mean IP score (Clance 16 item scale) was 57.93, SD 6.96. No significant sex differences in mean impostor tendencies. Impostor tendencies are positively related to neuroticism, maladaptive perfectionism and negatively to conscientiousness and adaptive perfectionism. No significant relationships were observed between impostor tendencies and openness or agreeableness. Significant moderation effects on were found in the present study for job satisfaction and organizational citizenship behavior.
Villwock et al. (2016)(73)	USA	To assess gender and other demographics associated with IP and evaluate weather IP is associated with burnout	Medical Students	138 RR – 5.28%	56%	18-24yrs - 43%, 25-30yrs - 50%	Cross sectional survey	Prevalence: 49% of females and 23.7% of males displayed IS (Young Imposter Scale). White and Asian race/ethnicity is associated with lower rates of IS (30%) compared with other races (73%). Female gender was not significantly associated with burnout. Students with IS had increased levels of exhaustion, emotional exhaustion, cynicism, and depersonalization. Fourth year students had higher level of IS than other years.
Want & Kleitman (2006) (74)	Australia	To examine parental rearing styles and objective confidence in relation to impostor phenomenon and self-handicapping tendencies	Mixed occupations including doctors, lawyers, executives, graduate students, small business owners	115 RR – not stated	62%	39yrs	Cross sectional survey	Prevalence: Not Stated; Mean IP score (Clance 20 item scale) was 53.61, SD 13.22. Self-handicapping tendencies correlate positively with feelings of impostorism. Impostor scores correlate negatively with paternal care/warmth, they show no significant correlation with maternal care/warmth. Impostor scores correlate positively with both maternal and paternal overprotection scores. A similar pattern of correlations is evident for the Self-Handicapping Scale, yet there are significant negative correlations between self-handicapping and both parental care/warmth scores. Higher impostor scores correlate with lower confidence levels, but not with the accuracy score of the test. There are no significant correlations between age and either self-handicapping or imposter feelings.

^9–74^]

### Study Characteristics

**Figure 2:**
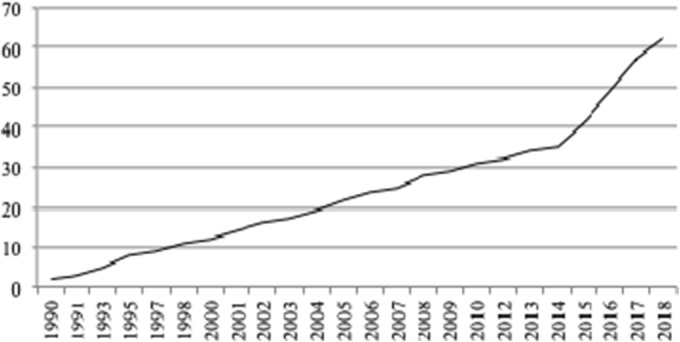
Included studies by publication year. Presents the distribution of the included studies by year of publication.

Although our searches were for the literature starting in 1966, the included studies were all published between 1990 and 2018 (Fig. 2)—notably, half were published in the past 6 years.

Although most of the studies were conducted in the USA^9, 10, 12, 13, 15–17, 21–32, 34–38, 42, 43, 45, 49–57, 61, 62, 64, 65, 67, 73^ and Canada,^19, 44, 46, 66, 68^ twenty-one studies evaluated populations in other countries including five in Austria,^39, 58–60, 63^ five in Australia/New Zealand,^20, 31, 70, 71, 74^ four in Germany,^11, 14, 47, 48, 58^ three in Iran,^33, 40, 41^ two in the UK,^20, 69^ and one each in Belgium^72^ and Korea.^73^

Nearly all of the included studies were single-arm observational studies (Table 1). Most commonly, the authors identified a population of interest, screened them with a validated impostor syndrome questionnaire and other psychometric assessment tools, then described the prevalence of impostor syndrome and co-occurring psychological issues. Two studies also included semi-structured interviews.^37, 38, 44^ One study included an experimental design in which subjects were exposed to successes and failures and asked to report on impostor feelings after these exposures.^31^ The only longitudinal assessment was of college students with impostor syndrome who were followed before and after a midterm examination.^25^ Notably, there were no randomized trials and only one study presented qualitative information about the clinical management people with impostor syndrome.

Overall, the quality of the included studies was fair: Only 20 studies reporting having Institutional Review Board (IRB) approval.^10, 12, 13, 20–23, 28, 44, 46, 48, 50–52, 54, 58, 59, 62, 64, 66, 73^ Many studies lacking IRB approval were of student populations—often in the authors’ own institutions. Response rates for the populations surveyed ranged from 2.8 to 97.2% (and many articles did not report response rates) (Table 1).

### Participant Characteristics

**Figure 3:**
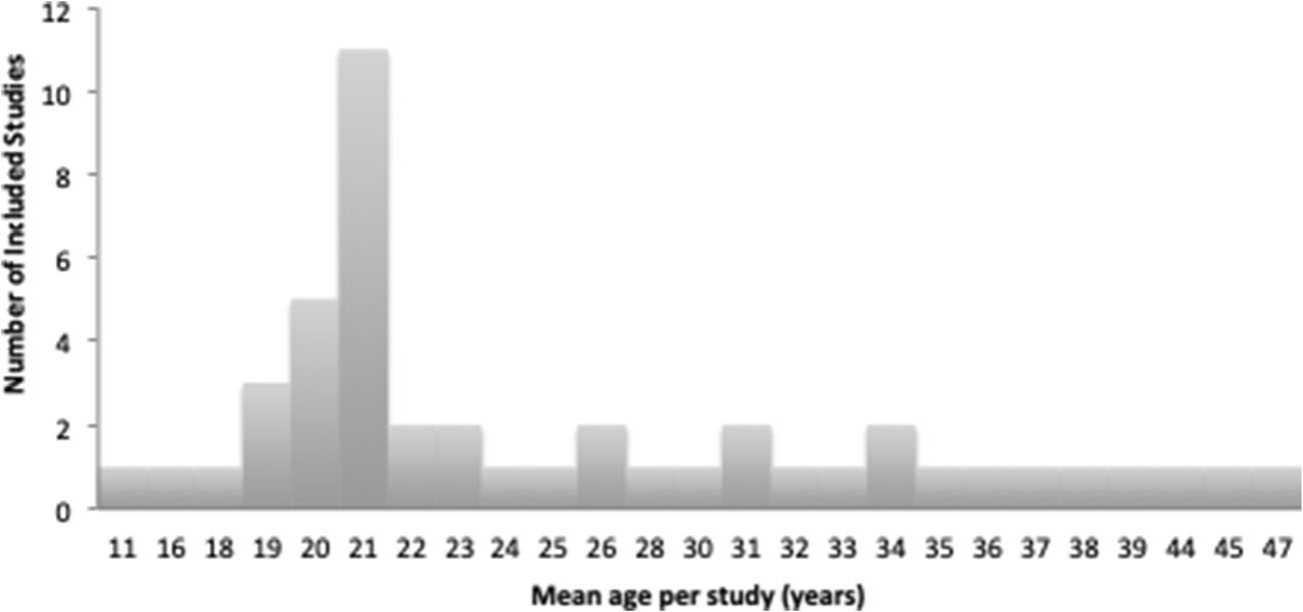
Distribution of mean age among the included studies. Presents the distribution of mean age for the included studies.

The included studies evaluated 14,161 participants, 60% of whom were women. Among those studies reporting a mean population age, the weighted mean age was 20 years (Fig. 3)—not surprising given that 34 of the included studies were of students. However, 17 studies included populations with a mean age of > 30 years and 5 additional studies were of professional populations but did not report a mean age.]-->

#### Students

Half of the included studies were of student populations: 5 evaluated elementary and high-school students,^16, 19, 27, 49, 61^ 29 evaluated undergraduates,^10, 14, 20–25, 28, 30, 31, 35, 41–43, 45, 50–52, 55–58, 63–66, 68–70^ and 12 evaluated graduate students.^17, 29, 31, 33, 34, 36, 39, 50, 55, 65, 67, 73^ Students with impostor syndrome had fears that were significantly related to maintaining their social standing and not wanting to display imperfection to others^31^; however, social support and self-worth were highly negatively associated with impostor symptoms.^16^ Impostor feelings were significantly related to pessimism, perfectionistic traits, and low self-esteem, and although there were no differences in grades between impostors and non-impostors, impostors expected to perform worse and were more anxious about exams.^25^ Interestingly, Ferrari found that students with impostor feelings were significantly less likely to cheat on examinations and to plagiarize written assignments than students without impostor feelings.^30^

#### Students of Minority Groups

Eleven included articles evaluated impostor syndrome in minority groups.^10, 12, 13, 21–23, 29, 51, 52, 54, 64, 73^ They demonstrated that impostor syndrome is common among African American, Asian American, and Latino/a American college students and that impostor feelings are significantly negatively correlated with psychological well-being and positively correlated with depression and anxiety.^21–23, 54^ Several factors may predispose minority students to increased psychological stress during their educational experiences including lack of adequate financial aid, the need to work to support themselves in school, racial discrimination, enduring negative stereotypes, and being the first in their families to pursue advanced education.^21, 29^ Moreover, using MANOVA, one study found that impostor feelings were stronger predictors of mental health than minority status stress.^21^ Austin et al. found that impostor syndrome, depression, and survivor guilt were highly correlated among African American college students.^10^ Bernard et al. found that African American freshmen who reported frequent racial discrimination but low levels of distress from discrimination had higher levels of impostor syndrome than those who reported high levels of distress from racial discrimination.^12, 13^ Few non-White individuals have been included in the samples used to standardize the assessment for impostor syndrome—thereby potentially invalidating these tools for minority populations.^29^

#### Employed Populations

Nineteen of the included articles described impostor syndrome among employed populations.^9, 11, 14, 15, 26, 32, 37, 38, 40, 44, 46–48, 53, 58, 59, 62, 72, 74^ Five of these were of nurses and physicians,^9, 40, 44, 46, 62^ the rest included managers, teachers, and accountants, among others. Given the tendency of people with impostor syndrome to aggressively pursue achievement while not being able to accept recognition when success is achieved, affected employees may experience increased levels of stress, burnout, and decreased job performance and satisfaction over time.^26, 37, 72^ Employees who persistently question their professional legitimacy are at higher risk for experiencing adverse psychological outcomes with implications to career retention, advancement, and job performance. Moreover, impostor feelings among employees is associated with fear of failure, fear of success, and low self-esteem.^58^ Employees who report more impostor feelings report less career planning and motivation to lead.^58, 60^

Bechtoldt found that supervisors across a variety of industries scoring high on impostorism were more inclined to delegate both routine and challenging tasks to subordinates who doubted their own professional abilities.^11^

Crawford et al. found a significant relationship between impostor syndrome and self-reported conflict managing work/life balance among community college employees; however, this relationship was minimized if employees perceived greater organizational support.^26^ This suggests a role for managers and executives in mitigating the effects of impostor syndrome on employees.

Together, these findings suggest that individuals who struggle with impostor syndrome may be limited in their ability to fully develop their professional potential and may be a significant contributor to burnout both among healthcare professionals and others.

### Diagnostic Tools

Several tools have been developed to assess impostor syndrome. The preponderance of included articles used the Clance Imposter Phenomenon Scale (or its German translation),^75^ a 20-item scale on a 5-point Likert scale related to self-assessed competency, praise, and success. Six studies^16, 27, 29, 45, 49, 61^ used the Harvey Impostor Phenomenon Scale (HIPS),^76^ a 14-item questionnaire on a 7-point Likert scale regarding personality traits (where a higher score indicates greater identification with impostor syndrome). Two used the Leary Imposter Scale,^55, 56^ two used self-developed questionnaires,^15, 19^ one^45^ used the Perceived Fraudulence Scale,^1^ and one used the Young Imposter scale.^73^

There is considerable variation in how researchers interpret specific scores on the impostor syndrome diagnostic scales. For some, a score of < 40 on the CIPS denotes no impostorism, 40–59 mild, 60–79 moderate, and > 80 as severe impostor feelings.^75^ Others recommend using a score of 62 on the CIPs^77^ or use the median CIP score in their population^25^ to distinguish impostors from non-impostors. We note the cutoffs used, when reported.

### Prevalence

Few of the included studies were designed to assess the prevalence of impostor syndrome, which varied widely from 9 to 82% largely depending on the screening tool and cutoff used to assess symptoms. For example, Chae et al.^18^ found that the prevalence of impostor syndrome among 654 Korean Catholics varied from 24% using a CIPS cutoff of 62 to 39% using a cutoff of 58. We suspect that the included literature on the prevalence of impostor syndrome may be subject to publication bias (i.e., the tendency of journals to publish studies with positive findings rather than negative findings) since all of the included studies reported some participants endorsing impostor feelings.

### Predictors of Impostor Syndrome

#### Gender Effects

Thirty-three articles compared the rates of impostor syndrome by gender.^10, 14, 18, 21, 23, 25, 27, 28, 32, 35–39, 42, 43, 45–50, 54, 57, 62, 63, 68, 70, 72, 73^ Sixteen of these found that women reported statistically significantly higher rates of impostor feelings than men.^23, 28, 33, 35–39, 42, 43, 46, 50, 57, 62, 63, 73^ Hutchins and colleagues found that men and women cope differently with their impostor feelings.^37, 38^ In contrast, 17 studies found no difference in rates of impostor syndrome between men and women.^10, 18, 21, 24, 25, 27, 32, 40, 45, 47–49, 53, 54, 68, 70, 72^ Brauer and Proyer studied two populations—psychology students and professionals—and found gender effects for impostor syndrome only among the students, not the professionals.^14^ Thus, the body of evidence suggests that while impostor syndrome is common in women, it also affects men.

#### Age Effects

Six studies compared the rates of impostor syndrome by age.^14, 18, 49, 62, 70, 74^ Two studies reported that increased age was associated with decreased impostor feelings.^18, 70^ Three studies found no age effect.^49, 62, 74^ Brauer and Proyer evaluated impostor syndrome in two cohorts (244 psychology students and 222 working professionals in Germany)—they found that age was significantly negatively correlated with impostor feelings among working professionals but not undergraduates.^14^ Notably, in their study, the age range of the working professionals was much larger than that of the students, perhaps contributing to the likelihood of finding an age effect.

### Comorbid Conditions

Many included articles explored the psychological issues that are often found to co-exist with impostor syndrome including depression,^41, 47–49, 57, 62, 69, 78^ anxiety,^34, 41, 79, 80^ low self-esteem,^58, 62^ somatic symptoms, and social dysfunctions.^41^ Impostor feelings among high school students correlated significantly with a history of prior suicidal ideation and attempts and depression.^49^ Clearly, the care of patients with impostor syndrome requires a careful assessment for comorbid conditions and treatment of them in addition to addressing the impostor feelings.

### Treatment

None of the included articles presented an evaluation of a specific treatment (e.g., cognitive behavioral therapy) for managing impostor symptoms. A 1985 paper by Matthews and Clance qualitatively described their experiences in private practice caring for 41 people with impostor feelings.^53^ They recommended validating patients’ doubts and fears, directly addressing fears of failure, and providing group therapy since these patients often feel isolated and that they alone experience impostor feelings; however, no data were presented on treatment intensity, duration, or improvements on any diagnostic tool.

### Lay Literature Results

We found considerable lay interest in impostor syndrome. During the year (March 28, 2018–March 18, 2019), 2317 Internet articles were published on impostor syndrome (150–200 articles/month). These resulted in 133,425 engagements (e.g., “likes,” re-postings, comments) on social media platforms such as Facebook and Twitter. While numerous Internet users interacted with content originating from sites like Psychology Today (968 engagements), the majority of readers engaged with articles posted on blogging platforms like Medium.com (3111 engagements) or sites targeted toward professionals, like Inc.com (1568 engagements), LinkedIn.com (795 engagements), and Forbes.com (688 engagements).^81^

A detailed review of the content of these articles is outside the scope of this study. However, the vast majority were tagged as “What is…” articles, which define impostor syndrome followed by “How-To” articles, which offer treatment tips. Many of the articles classified as “What is…” articles also include tips about how to manage impostor syndrome. These tips run the gamut from “embracing authenticity” to “comparing notes with peers and mentors about shared impostor feelings”; however, much of this advice involves changing the thought processes that affirm feeling like a fraud. In 2018, Time Magazine published an article with an accompanying short video entitled, “Yes, Imposter syndrome is real. Here’s how to deal with it.”^82^ Among other suggestions, they recommend that people suffering with impostor syndrome learn to reframe their thoughts and visit a psychologist.

Notably, whereas the academic literature on the topic is nearly all indexed with the term *Impostor Phenomenon*, the entirety of the lay literature is indexed with the term *Imposter Syndrome*.

## DISCUSSION

This, the first published systematic review of the literature on impostor syndrome which includes a novel, if somewhat unconventional lay literature review, has six key findings. First, we found a large peer-reviewed literature of more than 60 studies, half of which were published in the last 6 years. This is congruent with the recent explosion in interest on the topic of impostor syndrome in the lay literature. There are several gaps between the peer-reviewed and lay literatures including that academics prefer the term Impostor Phenomenon while lay authors use Imposter Syndrome. Whereas the published literature included no studies of interventions to treat impostor syndrome, the lay literature abounds with advice on how to manage impostor symptoms.^83, 84^ Given the current state of the peer-reviewed literature, mental health professionals faced with patients suffering from impostor syndrome will likely use evidence-based treatments for comorbid conditions such as cognitive behavioral therapy for depression and anxiety, but do not have an evidence base upon which to rely specifically for the impostor symptoms. This is a critical gap in the published literature—we recommend a prospective evaluation of the use of individual and group cognitive behavioral therapy focused on addressing impostor feelings on clinical and workplace outcomes for employed populations across a range of professions. Moreover, we recommend that impostor syndrome be considered for inclusion in the Diagnostic and Statistical Manual of Mental Disorders (DSM) so that the approach to patients with these symptoms can be codified for behavioral health providers.

Second, much of the earliest literature on impostor syndrome focused on women. While women do suffer from impostor syndrome, half of the included studies that reported evaluating a gender effect found no difference in the rates of men and women suffering from impostor syndrome. An implication of this finding is that clinicians and employers should be alert for impostor feelings in their entire population, not just women.

Third, numerous studies found impostor syndrome to be prevalent among ethnic minorities. A key finding from one of these studies is that impostor syndrome is a stronger predictor of mental health issues than minority status stress. This is particularly significant given that research on ethnic minority populations tends to focus on their minority status and presumed experiences of discrimination, rather than the individual differences within a minority group such as the impostor syndrome. Another important finding is that attempts to standardize impostor syndrome assessments typically include small numbers of ethnic minorities, which raises questions of whether current impostor measures are valid for ethnic minority populations.^85^

Fourth, it would be reassuring to believe that impostor symptoms decline with age. Unfortunately, half of the six studies that reported on age effects found that impostor symptoms decline with age but half did not. Clearly, this is a key open question that future studies evaluating employed populations (rather than just evaluating students) could address.

Fifth, depression and anxiety are frequently comorbid with impostor feelings. In the absence of specific treatment recommendations for impostor syndrome, patients with impostor feelings should be rigorously screened for depression and anxiety and treated for these with evidence-based therapies. Individuals experiencing impostor syndrome often perceive themselves to be the “only one” having these feelings, resulting in even greater isolation.^53^ Thus, referral to group therapy in which peers/coworkers discuss their feelings of doubt and failure might be particularly therapeutic. Clinicians and other high-achieving professionals may be reluctant to participate in such groups unless they are carefully designed to normalize and destigmatize impostor feelings and provide a safe environment in which to share experiences openly.

Finally, there is robust literature that describes the harmful association between impostor feelings and job performance, job satisfaction, and burnout among various employee populations, including healthcare professionals. In light of this evidence, we encourage professors and employers to incorporate recognition of this phenomenon in the development of both structured (e.g., training, orientation, onboarding) and unstructured (e.g., mentoring, coaching, self-directed learning) learning and career development activities. Success-oriented employees such as clinicians commonly have a thirst for training and personal growth. Offering resources such as access to therapy and resilience trainings that focus on impostor syndrome could help reduce the prevalence of impostorism in employed populations. When applicable, de-identified assessments can help the employees evaluate their personal change over time, while enabling managers to assess the impact of the structured intervention. In addition to structured and unstructured learning, professors and employers can target impostor syndrome by creating healthier expectations and a culture where mistakes are not interpreted as failures and publicly acknowledging and celebrating employee accomplishments.

Our study reflects the limitations of the included studies. First, nearly all studies were of the same design (cross-sectional surveys, often of convenience samples). The literature suffers from a lack of randomized and prospective trials and is likely subject to publication bias. Second, the weighted mean age of the studies that reported the age of their population was 20 years old, a finding driven by the preponderance of student populations among the included articles. However, given that 19 of the included studies described impostor syndrome in employed populations, the results of this review are relevant to older populations as well. Third, few studies addressed clinicians outside of academic environments—a critical gap given burnout among clinicians in a wide range of clinical settings. Additionally, the included studies evaluated workers only in a limited number of professional settings—none evaluated workers in technology companies—an important limitation given the amount of lay literature specifically targeting this population. Finally, given the nature of the reported data, we were precluded from performing quantitative synthesis of the included studies.

Our study is the first published synthesis of the peer-reviewed evidence on impostor syndrome. Our results suggest that impostor symptoms are prevalent among men and women and members of multiple ethnic groups, and are significantly associated with worsened experiences both in academic and professional settings. The literature critically lacks thoughtful evaluations of treatments of impostor feelings. We encourage caregivers, professors, and employers to be mindful of the likelihood of impostor syndrome in the populations under their care and to take steps to mitigate these feelings.

## Appendix

### Search Terms

The following search terms were used to identify articles indexed between January 1966 and May 2018. We did not limit by language publication type or other terms

- Imposter Syndrome*
- Imposter Phenomenon*
- Impostor Syndrome*
- Impostor Phenomenon*
- Imposter Phenomenon AND/OR Imposter Syndrome
- Impostor syndrome AND/OR Impostor phenomenon*
